# Impacts of tropical cyclones on food security, health and biodiversity

**DOI:** 10.2471/BLT.22.288838

**Published:** 2022-12-01

**Authors:** Andrea Monica D Ortiz, Paul L C Chua, Dante Salvador, Cecilie Dyngeland, Jose Dante G Albao, Rene A Abesamis

**Affiliations:** a Instituto de Ecología y Biodiversidad, Universidad de Concepción, Victoria 631, Barrio Universitario, Concepción 4070374, Chile.; b Department of Global Health Policy, University of Tokyo, Tokyo, Japan.; c Institute of Social and Preventive Medicine, University of Bern, Bern, Switzerland.; d Department of Agricultural Sciences, Inland Norway University of Applied Sciences, Blæstad, Norway.; e Philippine Reef and Rainforest Conservation Foundation Inc., Cauayan, Philippines.; f Silliman University-Angelo King Center for Research and Environmental Management, Dumaguete City, Philippines.

Food security, human health and well-being largely depend on biodiversity. Biodiversity supports agriculture through ecosystem services such as pollination and water purification, and provides access to natural medicines, which are the primary source of health care for 4 billion people worldwide.^1^ However, climate stressors exert significant pressure on terrestrial and marine ecosystems.^1^^,^^2^ Climate change causes increases to temperature and changes to precipitation patterns. Importantly, it will also cause changes to the frequency and intensity of extreme events globally.^3^

Tropical cyclones, also known as typhoons or hurricanes,^4^ are among the most destructive natural hazards that threaten the nexus of food–health–biodiversity. Between 1998 and 2017, tropical cyclones killed 233 000 people and affected an estimated 726 million people worldwide.^3^ While recent analyses show that between 1990 and 2020 the annual number of tropical cyclones has declined, losses and damages have significantly increased, likely due to population growth and higher value of coastal assets.^5^ Because of climate change, tropical cyclones are projected to become more powerful, with storm surges causing higher levels of inundation because of sea-level rise, as well as higher rates of associated precipitation (Box 1).^6^

Box 1 **Tropical cyclones**

Tropical cyclones, also known as hurricanes or typhoons, are rapid rotating storms that develop over tropical oceans. They are called hurricanes when they occur in the Atlantic Ocean and the eastern north Pacific Ocean, typhoons in the western Pacific Ocean, or cyclones in the Bay of Bengal and Arabian Sea.^4^

Every year, about 45 tropical cyclones form worldwide.^4^ These storms bring violent winds (exceeding 119 km/h), torrential rain, high waves and, in some cases, destructive storm surges and coastal flooding. While their powerful winds can cause massive losses and damages, the greatest damage to life and property is from events such as storm surges, flooding, landslides and tornadoes.^3^

Research indicates that climate change will increase the global tropical cyclone risk because of the likely increase in the proportion of intense tropical cyclones. Most studies project a decrease in the global frequency of tropical cyclones with warming, although with a large range of uncertainty that includes the potential for increases. ^6^

Tropical cyclones occur in areas where large concentrations of people and infrastructure exist – as well as poverty and inequalities that contribute to countries’ vulnerability to climate risks. As biodiversity is highest around the tropics,^2^ tropical cyclones and their associated hazards affect not only people and assets, but also species and ecosystems that support livelihoods, services and products for billions of people. Hence, weather extremes such as tropical cyclones have important short- and long-term impacts on food security, health and nutrition, and biodiversity. 

However, much remains to be understood about the range of ecological impacts of tropical cyclones; researchers and policy-makers need to give more importance to the role of biodiversity in food systems, health and nutrition.^7^^,^^8^ Here, we discuss how tropical cyclones affect the food–biodiversity–health nexus and highlight future research and policy opportunities.

## Food security

Because tropical cyclones destroy crops, cropland and terrestrial and marine ecosystems, food security is strained in their immediate aftermath. Many rural, coastal and indigenous communities depend on the access to wild and traditional foods rich in vitamins and minerals provided by tropical forests and coral reefs. Defoliated tree canopies, increased wood debris and decimated coral caused by tropical cyclones therefore affect food security, especially for poor households with fewer income-generating activities.^9^^–^^11^

In the long-term, the destruction of roads, agricultural infrastructure, grazing lands and assets can reduce production capacity, including livestock farming and farm-to-market connectivity. This impact can decrease the food supply and disrupt the supply chain. Furthermore, climate-related disasters are known contributors to civil tensions, forced migration and even conflict.^12^

Agricultural land can also become infertile over extended periods of time due to vegetation loss and coastal erosion caused by storm surges, violent winds and saltwater intrusion. Affected tropical forests will also alter the habitat of species that support agriculture and provide less income from forestry activities.^8^^,^^13^ Coastal land use and land cover (such as coastal vegetation, built areas and other man-made infrastructure) can modulate or amplify the extent of devastation caused by tropical cyclones.^14^

## Health and well-being

Tropical cyclones threaten the health and well-being of exposed and vulnerable populations. For example, flash floods and storm surges can cause drowning and physical trauma, including from hazardous materials and exposed electrical wiring. Furthermore, floodwater and contaminated water supplies increase the likelihood of acquiring water- and vector-borne infectious diseases. 

The indirect health impacts include disruptions to health systems and services by damaging basic infrastructure, food and water supplies, and safe shelter. These losses and damages can exacerbate strained health systems. For example, tropical cyclones that occurred concurrently with the coronavirus disease 2019 (COVID-19) pandemic resulted in a surge in COVID-19 cases and increased reports of poor mental health in the Philippines.^15^

### Nutrition and ecosystems

Tropical cyclones affect nutrition – a key component of health – because of the devastating effect such storms can have on food security. For example, pregnant mothers and their young children had poor nutritional and relative weight status several years after cyclone Aila in India in 2009.^16^

Tropical cyclones also affect another important, albeit less visible, aspect of nutrition, which is micronutrient adequacy. Around 2 billion people worldwide have micronutrient deficiency, and this hidden problem has lifelong consequences on health.^5^ Tropical ecosystems providing nutritious food are the most susceptible to the combined effects of tropical cyclones, climate and land use change. Changes to the frequency and magnitude of tropical cyclones, in combination with other human disturbances, are leading to unprecedented negative ecological consequences for tropical forests and coral reefs, which in turn negatively affect human nutrition.^2^ Micronutrient-rich tropical reef fish contribute significantly to nutrition and dietary quality where there is high consumption;^17^ therefore, the destruction of coral reefs by tropical cyclones can have serious consequences for coastal communities.

## The way forward

### Research

Considerable evidence exists that tropical cyclones affect the nexus of food–biodiversity–health. We hypothesize that the changes in tropical cyclone activity will increase these events’ negative impacts on tropical ecosystems, which are already seriously threatened by the combined impacts of human activities, land use change and climate change. Projected changes in tropical cyclones’ intensity will in turn have impacts on dietary diversity, an important indicator of the connections between food security, nutritional status and biodiversity.^10^ To help policy-makers develop evidence-based strategies to protect biodiversity, food security and health, quantifying the complex interactions and causal links of how tropical cyclones affect this nexus is needed in research. Modelling approaches can be used to link data on biodiversity (such as forest cover, coral reef extent, species richness and species abundance), tropical storm activity and health outcomes, to develop scenario-based projections of dietary diversity. However, while many factors of this complex nexus are known, more research and interdisciplinary collaborations are needed to identify the roles and impacts of socioeconomic and policy factors, such as trade and land use.^7^

### Policies

In the policy domain, greater coordination across the major frameworks on biodiversity, climate change and disasters can aid in the development of cross-cutting programmes between national governments. For instance, the Sendai Framework for Disaster Risk Reduction^18^ outlines four priority actions: (i) understanding disaster risk; (ii) strengthening governance; (iii) investing for resilience; and (iv) enhancing preparedness and recovery. These actions complement the United Nations Convention on Biological Diversity Post-2020 Global Biodiversity Framework,^19^ which outlines three domains of specific action targets for 2030: (i) reducing threats to biodiversity; (ii) sustainable use and benefit sharing; and (iii) using tools and solutions for implementation and mainstreaming. Giving prominence to the role of ecosystems and sustainable agricultural landscapes across these conventions, including for climate change, is needed to address the food–health–biodiversity nexus.^8^ Science-based targets for sustainable and healthy diets, and integrated approaches such as planetary health and One Health may be useful in this regard.

Biodiversity, which is essential to human well-being, is threatened by tropical cyclones; at the same time, meeting the sustainable development goals is impossible without the protection of biodiversity. Therefore, continued and enhanced support for programmes and policies for biodiversity protection, alongside robust food and health systems, is needed to increase the resilience of local people and nature in future scenarios of climate change and variability. These initiatives include local management actions, including ecological approaches to landscapes and agricultural production, to protect rural and indigenous communities’ access to forests and fisheries for wild and traditional foods, ecological functioning and biodiversity.^7^^,^^8^^,^^10^^,^^17^

The impacts of tropical cyclones on health and well-being depend on people’s exposure, their vulnerability and their characteristics. Factors such as governance, building design, early warning systems and disaster risk reduction and management plans are critical in mitigating the impacts resulting from a tropical cyclone.^3^

In addition, tropical cyclones cause many intangible social impacts. The destruction from tropical cyclones can affect cultural ecosystem services (that is, the non-material benefits people gain from ecosystems, such as spiritual enrichment, recreation and aesthetic experiences), people’s cultures and identities, and their relationships that are intertwined with nature. For instance, while the destruction of cropland can be measured, the loss of a sacred tree and other intangible losses caused by tropical cyclones cannot, and these may have profound impacts on people.^20^ Greater efforts are needed to understand and address intangible, social and personal losses in post-tropical cyclone recovery.

More intense tropical cyclone activity increases the risks of significant losses and damages to the nexus of food security, health and biodiversity. Greater synergy in both research and policy frameworks is needed to protect biodiversity and its contributions to people’s food security, health and well-being.
